# What Is the Best Pulmonary Physiotherapy Method in ICU?

**DOI:** 10.1155/2016/4752467

**Published:** 2016-04-24

**Authors:** Ufuk Kuyrukluyildiz, Orhan Binici, İlke Kupeli, Nurel Erturk, Barış Gulhan, Fethi Akyol, Adalet Ozcicek, Didem Onk, Guldane Karabakan

**Affiliations:** ^1^Department of Anesthesiology and Reanimation, Faculty of Medicine, Erzincan University, 24100 Erzincan, Turkey; ^2^Physiotherapy and Rehabilitation Clinic, Mengucek Gazi Education and Training Hospital, Erzincan, Turkey; ^3^Department of Microbiology, Faculty of Medicine, Erzincan University, Erzincan, Turkey; ^4^Department of Internal Medicine, Faculty of Medicine, Erzincan University, Erzincan, Turkey; ^5^Department of Anesthesiology and Reanimation, Faculty of Medicine, Mersin University, Mersin, Turkey

## Abstract

*Objective*. Effects of high frequency chest wall oscillation technique were investigated on intubated ICU patients.* Background*. Thirty intubated patients were included in the study. The control group (*n* = 15) received routine pulmonary rehabilitation technique. In addition to the pulmonary rehabilitation technique, the study group (*n* = 15) was given high frequency chest wall oscillation (HFCWO). APACHE II, dry sputum weight, lung collapse index, and blood gas values were measured at 24th, 48th, and 72nd hours and endotracheal aspirate culture was studied at initial and 72nd hour. The days of ventilation and days in ICU were evaluated.* Results*. There is no significant difference between APACHE II scores of groups. The dry sputum weights increased in the study group at 72nd hour (*p* = 0.001). The lung collapse index decreased in study group at 48th (*p* = 0.003) and 72nd hours (*p* < 0.001). The PO_2_ levels increased in the study group at 72nd hour (*p* = 0.015). The culture positivity at 72nd hour was decreased to 20%. The days of ventilation and staying in ICU did not differ between the groups.* Conclusions*. Although HFCWO is very expensive equipment, combined technique may prevent the development of lung atelectasis or hospital-acquired pneumonia more than routine pulmonary rehabilitation. It does not change intubated period and length of stay in ICU. However, more further controlled clinical studies are needed to use it in ICU.

## 1. Introduction

Pulmonary complications are the most common causes of morbidity and mortality in ICU patients [1]. Intensive care unit patients are usually intubated and monitored through mechanical ventilator. This makes it difficult to clean the airway passages of these patients [2]. The airway secretions, which cannot be excreted, have caused the transient mucociliary dysfunction and inability to cough. As a result of these secretions, intrapulmonary shunts can be developed and tidal volume has reduced. Finally complications extending to atelectasis, bronchopulmonary inflammation and infections, pneumonia, respiratory arrest, and even death can occur accordingly [3–5]. Several techniques and devices can be used in order to mobilize the secretions, make the coughing effective, and improve cleaning of the airway. The conventional chest physiotherapy is a method including postural drainage, position, and percussion technique [6]. Applied for many years and supporting the airway clearance, this method of airway clearance is human-dependent and frequency and duration of activity vary according to the practitioners. Likewise it has been reported that conventional method is labor intensive, with relatively low percentage of therapy and discomfort for patients [7].

High frequency chest wall oscillation (HFCWO) has been used for a long time on the treatment of chronic conditions such as cystic fibrosis, bronchiectasis, and neuromotor-neuromuscular disorders. HFCWO loosens the mucus adherent to the bronchial airway and creates a cough-like effect [8, 9]. It has an important advantage like making standard mechanical therapy without dependent practitioners [10]. HFCWO has been typically applied for a certain time in the standard mode with a vest attached to the air-blast generator [11].

In our literature searching whereas we found studies about tolerability, comfort, and pain levels of these two techniques, there were not any studies including, for example, dry sputum weight of intubated patients, development of lung atelectasis or hospital-acquired pneumonia, and days in ventilation and days of staying in ICU.

In this study, we aimed to evaluate the effect of HFCWO technique in addition to routine conventional pulmonary physiotherapy method particularly in ICU patients under the mechanical ventilator and to investigate its effects on the lung collapse and excretion of the airway secretions and correlation between the amount of excreted secretions, frequency of pulmonary infections, and the period of staying in ICU.

## 2. Material Method

The study was approved by the Erzincan University Medicine Faculty Ethical Assessment Commission for the Researches on Human (letter dated 18.02.2014 and numbered 26-1455), and the required ethical committee permit was obtained from patients' relatives. Patients with rib fracture, acute hemorrhage, unstable intracranial pressure, and existence of chest drainage tube and those having history of spinal surgery, skin infection in the back and chest area, and subcutaneous emphysema were excluded from the study.

### 2.1. Study Protocol

Strength of the study was calculated based on the study by Chen et al. in which chest vibration treatment applied together with the routine positioning care reduced to 48.4% of dry sputum weight after 72 hours [12]. Accordingly, sample size estimation showed that approximately 30 patients were needed to detect a clinically relevant reduction of pulmonary secretion, with a power of 0.91 and alpha of 5% by using a two-sided two-sample equal-variance *t*-test.

Patients with more than three days' intubation in critical care unit of Mengücekgazi Training and Research Hospital were included in this study. Settings of the mechanical ventilators were the same for all the patients and set at the pressure-controlled mode with 15–25 mmHg pressure, 8 mL/kg tidal volume, and PEEP: 3–10 cmH_2_O. Moisturizers at 37°C temperature and 100% humidity were used in all the ventilators. Heat and humidity level of the ventilator are checked every 12 hours. Respiratory sounds of the patients were evaluated and secretions were aspirated when it was deemed necessary.

We have only one high frequency chest wall oscillation machine (TheVest®, Model 205) and patients were divided into two groups of 15 patients according to the appropriateness of the HFCWO device.

*Group K*. This was named as the control group receiving routine pulmonary rehabilitation consisting of position giving technique (left lateral, supine, and right lateral), chest wall percussion, and airway aspiration at every 3 hours and in addition to this rehabilitation postural drainage was used if patients were extubated.

*Group S*. This group was called study group given HFCWO technique for 72 hours in addition to the routine pulmonary rehabilitation method. The technique was applied with 7–10 hz frequency given by a ped wrapped around the thorax and pulmonary rehabilitation with 3 mmHg pressure four times a day with 15-minute periods (TheVest, Model 205). It was terminated in the patients with cardiac apex beat > 20/min, respiratory rate > 10/min, >20 mmHg decrease in the mean arterial pressure, and SpO_2_ < 95% during the vibration.

Each patient's age, gender, diagnosis of the disease, and initial APACHE II scores were recorded. The arterial blood gas, PA chest X-ray, dry sputum weight, and lung collapse index (LCI) were evaluated at initial, 24th, 48th, and 72nd hours. Endotracheal aspirate culture was evaluated in the samples collected at initial period and 72nd hour. In addition, days in ventilation and days of staying in ICU were calculated.

Lung collapse index (LCI) values were recorded by listening to both lungs and scoring between 0 and 4 (0: normal expansion, 1: single lobe collapse, 2: 2-lobe collapse, and 3: multiple lobe collapse) by two experienced anesthetists. When the scores of the same hour were given different by them, the consensus was obtained by coming together and discussing face to face. In order to determine dry sputum weight (DSW), aspiration samples collected from the patients were put into 50 mL falcon tubes and centrifuged at 3500 rpm for 30 minutes by blind laboratory assistant, supernatant fluid was poured, and the remaining mucous portion was weighed. Dead weight of the falcon tubes was subtracted from the values obtained and weight of the sputum was found as gram (gr).

### 2.2. Statistical Analysis

Statistical analyses were carried out using the Statistical Package for Social Sciences, Windows, version 20.0 (SPSS, Chicago, IL, USA). Descriptive statistics for each variable were determined. Normality of the data distribution was assessed with the Kolmogorov-Smirnov test. Results for continuous variables were demonstrated as mean ± standard deviation. Results for continuous variables without normal distribution were demonstrated as median (min–max). Statistical significant differences between the groups were determined by chi-square test for categorical variables. For continuous variables, nonparametric statistics (Mann-Whitney *U*) and parametric statistics (*t*-test) were all used, as appropriate. Associations between the variables were explored using Spearman's rho (for data that was not normally distributed).

## 3. Results

This study included a total of 30 patients with 15 patients in the study and 15 in the control group.

Of the cases included, 63.3% (*n* = 19) were male and 36.6% (*n* = 11) were female patients. Of the study group, 66.6% (*n* = 10) were male and 33.3% (*n* = 5) were female patients. Of the control group 60% (*n* = 9) were male and 30% (*n* = 6) were female patients. No significant difference was observed between the groups in terms of the patients' gender (*p* > 0.05).

The mean age of the patients was found as 73.1 ± 11.9 in Group S and 74.7 ± 11.5 in Group K and there were no statistically significant differences between the groups (*p* = 0.712).

APACHE II score of Group K was 27.9 ± 7.4 and 26.5 ± 6.1 for Group S. According to these results there was no difference between the groups in terms of APACHE II scores (*p* = 0.584).

Median, minimum, and maximum DSW amounts and LCI scores of the study and control groups are given in Table 1. Despite the fact that statistical differences between the groups in terms of DSW were not significant at the 24th and 48th hours, decrease at the 72nd hour was found to be statistically significant (*p* = 0.001).

No significant difference was observed between the groups in terms of LCI at initial period and 24th hour, while a statistically significant decrease was seen at 48th hour (*p* = 0.003) and 72nd hour (*p* < 0.001) (Table 1).

The PO_2_ and lactate levels of the control and study group are shown in Table 2. There was no statistically significant difference between the groups in terms of PO_2_ at 0, 24, and 48 hours, while a significant elevation was found in the study group at 72nd hour (*p* = 0.015).

No significant difference was found between the groups in terms of lactate at 0 and 48 hours; however there was a statistically significant increase in the study group at the 24th hour (*p* = 0.015) and a significant decrease at the 72nd hour (*p* = 0.033).

Table 3 shows the patients' processes in ICU. There are no statistically significant differences between groups for intubated period of patients and days of staying in ICU (*p* > 0.05).

The initial endotracheal culture positivity was 13.3% and at the end of 72nd hour was 53.3% in Group K. We found that culture positivity was decreased to 20% at the 72nd hour compared to initial culture results for Group S in the endotracheal culture material (Table 4).

In addition to these results, the correlation between LCI and DSW was evaluated with Pearson correlation test and a significant correlation was found at the end of the 72nd hour (*r* = 0.471; *p* = 0.009).

## 4. Discussion

The function of airway cleaning decreases in the intubated patients. Their mucociliary clearance has impaired and the risk for lung collapse has increased. Therefore, the secretion retention, atelectasis, and ventilator related pneumonia are often observed, respectively, in ICU patients. Several airway cleaning techniques and pulmonary rehabilitation approaches are used to prevent atelectasis increasing susceptibility to pulmonary infection like ventilator-associated pneumonia. Increased air flow-mucus interaction, decreased mucus viscoelasticity, and elevated ciliary activity during HFCWO application are the physiologic mechanisms providing cleaning of the airway [8, 13]. HFCWO application both has been demonstrated to improve mucociliary cleaning and has increased central or peripheric mucus cleaning by causing a temporary increase in expiratory flow and production of cough-like shear forces and decrease in the mucus viscoelasticity [9, 14–16].

In our study DSW was gradually increased in the control group at 48th and 72nd hours; on the contrary it was gradually decreased in the study group given HFCWO and the amount at the 72nd hour was found to be statistically significant. LCI values were gradually decreased in the study group at 24th, 48th and 72nd hours; furthermore statistically significant decrease was found at 48th and 72nd hours. The reductions of DSW amounts and LCI scores in study group indicate that the secretions were excreted more easily within hours and days. Moreover these reductions led to lower frequency of monitoring pulmonary obstructions or atelectasis in study group rather than control patients. Our results were consistent with the previous studies in the literature [12, 16–19].

In a study of 29 hospitalized patients with cystic fibrosis patients comparing HFCWO to conventional chest physiotherapy, no statistically significant change in pulmonary function testing or oxygen saturation was observed after either HFCWO or CCPT compared with baseline [20]. As Allan et al. found stable pulse oximetry and haemodynamic values, the PO_2_ values evaluated in the arterial blood gas were changed statistically insignificantly at 24th and 48th hours in our study [21]. But in the study group only at 72nd hour was an enhancement determined statistically significant. Meanwhile there were statistically insignificant decreases in PCO_2_ values in the study group at 24, 48, and 72 hours. PO_2_ value which was found to be significant in the study group at the end of 72nd hour and gradually decreased PCO_2_ values at 24th, 48th, and 72nd hours indicate that the vibrations in the chest increased secretion drainage in the airway and enabled easier excretion of the secretions, resulting in the cleaning of airway which in turn affected the blood gas values. Our results were consistent with those of the previous studies [22, 23].

Clinkscale et al. suggested that conventional chest physiotherapy and HFCWO may be equivalent in terms of their effects on patient outcomes [24]. Although one of Group S patients and two of Group C patients were exitus in our study, the days of ventilation and staying in ICU did not differ between the groups. These outcomes were consistent with Clinkscale et al.

The culture positivity was found as increased by 40% in the control group and decreased by 20% in the group given HFCWO at the end of 72nd hour. Furthermore a significant correlation was detected between LCI and DSW at the end of 72nd hour. It was thought that these findings are similar to literature [12].

Because of more easily excreted secretions the cleaning of the airway was provided considerably. Hence, the results may be interpretable as decreasing frequency of lung atelectasis or hospital-acquired pneumonia. Whereas these findings pointed out the advantages of combined technique, the lack of significant difference between groups with regard to number of days in ICU and intubated number of days may question the effectiveness of this device in prognosis of critically ill patients.

## 5. Limitation

Some several potential limitations of our study should be noted. First, it was a single center study, limiting the generalizing ability of the results, and we had a heterogeneous patient population. Secondly although we calculated the numbers of patients by power analysis, we think that more samples are needed for more acceptable results for patient outcomes or mortality.

## 6. Conclusion

Many physiotherapy methods are used both to prevent pulmonary dysfunction and to make early extubation. But we can suggest that using HFCWO in addition to the routine physiotherapy methods can provide more secretion mobilization through the lungs in ICU. Thus, it can be possible to prevent the development of lung atelectasis or hospital-acquired pneumonia. Although applying HFCWO together with routine physiotherapy methods may be more effective method to prevent atelectasis and pulmonary infections, we need not forget that HFCWO may be unavailable for some hospitals because of its cost. However, more further controlled clinical studies are needed in order to use this knowledge to ICU practices.

## Figures and Tables

**Table 1:** Demographic, DSW, and LCI data.

	Group C	Group S	*p* value
Age	73.1 ± 11.9	74.7 ± 11.5	0.712^*∗*^
Apache II	27.9 ± 7.4	26.5 ± 6.1	0.584^*∗*^
DSW 24th hour	4.9 (0–19.8)	8 (0.9–40.2)	0.187^*∗∗*^
DSW 48th hour	6.5 (0.8–23.2)	5.3 (1–30.2)	0.512^*∗∗*^
DSW 72nd hour	11.2 (4–32.1)	2.5 (0–18.2)	0.001^*∗∗*^
LCI initial hour	2 (1–3)	3 (0–3)	0.233^*∗∗*^
LCI 24th hour	2 (1–3)	2 (0–3)	0.116^*∗∗*^
LCI 48th hour	3 (2-3)	2 (0–3)	0.003^*∗∗*^
LCI 72nd hour	3 (1–3)	1 (0–2)	<0.001^*∗∗*^

^**∗∗**^Mann-Whitney *U* (data are given as median (min–max)).

^**∗**^ *t*-test (data are given as mean ± SD).

DSW: dry sputum weight. LCI: lung collapse index.

**Table 2:** Blood gas analysis.

	Group K	Group S	*p* value
PO_2_ initial hour	83.6 (38.9–231)	87.4 (59.9–140)	0.713^*∗∗*^
PO_2_ 24th hour	84.4 (42.4–156)	90 (58–137)	0.713^*∗∗*^
PO_2_ 48th hour	79.8 (40.8–184)	85.2 (70.2–129)	0.098^*∗∗*^
PO_2_ 72nd hour	70.4 (43.8–121)	88.1 (69–153)	0.015^*∗∗*^
Lactate initial hour	1.7 (1–7.4)	1.4 (0.7–2.6)	0.061^*∗∗*^
Lactate 24th hour	1.9 (1–8.1)	1.6 (0.9–3.2)	0.015^*∗∗*^
Lactate 48th hour	1.8 (0.8–9.2)	1.6 (0.9–4.7)	0.367^*∗∗*^
Lactate 72nd hour	1.7 (1.1–4)	1.5 (0.9–3.5)	0.033^*∗∗*^

^*∗∗*^Mann-Whitney *U* (data are given as median (min–max)).

*t*-test (data are given as mean ± SD).

**Table 3:** Patients outcomes.

	Group C	Group S	*p* value
Days in ICU	26 (4–87)	17 (8–51)	0.22
Intubated period	26 (4–87)	17 (6–51)	0.14

^*∗*^Mann-Whitney *U* (data are given as median (min–max)).

**Table 4:** The results of endotracheal cultures.

	Initial endotracheal culture		Endotracheal culture at 72nd hour	
	Positivity (+) patients	Negativity (−) patients	Positivity (+) patients	Negativity (−) patients
Group K	2 (13.3%)	13 (86.7%)	8 (53.3%)	7 (46.7%)
Group S	9 (60%)	6 (40%)	6 (40%)	9 (60%)
